# Editorial: State-of-art of 3D printing and bioprinting technology in various domains of biomedicine, tissue engineering and regenerative medicine

**DOI:** 10.3389/fbioe.2025.1703915

**Published:** 2025-11-07

**Authors:** Sudipto Datta

**Affiliations:** All India Institute of Medical Sciences, New Delhi, India

**Keywords:** bioprinting, 3D printing, hydrogel, bioink, tissue engineering

## Introduction

In the last 10 years, the methods of 3D printing and bioprinting have advanced dramatically, providing groundbreaking approaches to developing scaffolds, personalized implants, tissue constructs, organoids, and even therapeutic devices. The Research Topic “State-of-the-art of 3D Printing and Bioprinting Technology in various domains of Biomedicine, Tissue Engineering and Regenerative Medicine” compiles a wide-ranging and timely set of reviews, original research, and mini reviews that together capture present mastery, explain enduring problems, and delineate useful new pathways.

## Aims and scope

The Research Topic seeks to capture:

- Recent revelations in bioinks, scaffold design, multimaterials, bioprinting, and printing systems design technologies.
- Patient-specific implants, along with bioengineering and therapeutic uses such as bone, cartilage, tympanic membrane, skin, liver, and devices, as well as wound healing.
- Assessing translational and preclinical readiness *in vivo* biocompatibility, tissue vascularization, regulatory and scale manufacturability, as well as reproducibility.
- Advanced concepts and emerging paradigms such as 4D printing, custom GelMA or alginate/cellulose blend incorporated with decellularized matrices, and biologically active component addition.

## Contributions in the Research Topic and key insights

The Research Topic consists of ten articles (5–10 ⇒ max 1,000 words), incorporating empirical research, systematic reviews, and mini reviews. Organized by theme, below are the highlights.

### Technological and material innovations

- Grandjean et al. were able to produce an innovative hydrogel bioink from a mixture of alginate and cellulose and Thrombocyte concentrate for tissue regeneration in WAYS: Incorporating structural supports with biological growth factors. Towards optimized tissue regeneration: a new 3D printable bioink of alginate/cellulose hydrogel.
- Gaglio et al., along with Baruffaldi and Pirri, also evaluated the different origins and methods synthesized for the production of GelMA (gelatin methacryloyl) necessary for multimaterial bioprinting and synthesized valuable information that relates to the mechanical, rheological, and crosslinking print properties in addition to the biological compatibility of the materials used in the print.

### Biomedical applications

- The review “Bioprinting of gelatin-based materials for orthopedic application” by Waidi et al. discusses the state of gelatin and its derivatives for bone and cartilage constructs while pinpointing the challenges in mechanical strength, degradation kinetics, and integration.
- *In vivo* characterization by Laubach et al. of 3D-printed polycaprolactone-hydroxyapatite scaffolds with a Voronoi architecture shows an increase in scaffold-guided bone regeneration, confirming that scaffold biomimetic design and composite materials are progressively yielding relevant translational outcomes.

### Clinical and translational reviews

- In Application of 3D printing in the treatment of diabetic foot ulcers: current status and new insights, Li et al. describe the functionalities of 3D printing wound dressings, scaffolds, and possibly devices with an emphasis on diabetic foot ulcers, the associated healing and infection issues being onerous on the clinical area.
- In Advances in 3D printing for the repair of tympanic membrane perforation: a comprehensive review, Xue et al. summarize material alternatives, scaffold construction, and mechanical and acoustic features of a particularly important niche for the ear membrane’s functional requirements.
- In 3D printing and bioprinting in the battle against diabetes and its chronic complications, Sathisaran discusses the potential of additive manufacturing to tackle issues that extend beyond wounds, including the vascular, renal, and pancreatic complications associated with diabetes.

### Novel methodologies or concepts

- In the mini-review on iPSC-derived cells for whole liver bioengineering, Telles-Silva et al. suggest using cells derived from a pluripotent stem cell line for liver tissue engineering, which helps to address both cell sourcing and the complexity of organ engineering.
- Singh et al. Work on a caprine pancreas-derived extracellular matrix scaffold analyzes the decellularization methods (immersion vs. perfusion) designed to make scaffolds from emerging tissue banks–a reminder that it is not only printing, but the entire upstream biogenic material source, that counts.
- The review 4-Dimensional printing: exploring current and future capabilities in biomedical and healthcare systems by Agarwal et al. adds on to our understanding not only of printed static constructs, but also dynamic constructs which over time change in shape, material properties, or behaviour.
- In the same vein, Chang and Sun in Laser bioprinting in biomedicine: review of principles, techniques, and applications discuss advanced techniques of laser-guided bioprinting, promising cell-friendly, highly localized cell deposition, and biostructures of intricate geometry.

### Common challenges and overarching themes

From these contributions, certain recurrent challenges emerge:

1. The Challenges of Bioink Design: Striking a balance between optimal rheology, bioink printability, cell viability, bioink mechanical strength and degradability, and bioink biological cues is still a challenge. Numerous studies indicate trade-offs.
2. Addressing Vascularization and Functional Integration: For thicker tissues and organs, there are still critical hurdles in satisfying host integration, vasculature, immune compatibility, and hosting sufficient nutrient and waste transport.
3. Importance of Scaffold Architecture: Voronoi designs, multimaterial layering, porosity, and interconnectivity. All of these, the scaffold structural parameters deeply influence tissue ingrowth, overall mechanical performance, and more.
4. The Dilemma of Translation vs. Scale: More *in vivo* studies appear every day, yet many technologies remain in the animal preclinical phase. Considerable Research Topic still exist around reproducibility, cost, ethics, and lack of standardization.
5. Smart Constructs: Advanced bioinks and responsive materials are still in their preliminary stages, and 4D printing has yet to reach the more widespread clinical level.
6. Bioinks and Decellularization: The ECM and its various sources, as in the case of the caprine pancreas, use of decellularization techniques, and the blending of natural polymers such as gelatin and alginate with synthetic composites like PCL-HA are proving critical in biocompatibility and mechanical compliance.

### Broader context and future directions

Putting these findings in the broader biomedicine/regenerative medicine field:

- The transition from proof-of-concept to more rigorous preclinical validation will require additional time in development. The community will need to develop standardized protocols for characterizing bioinks, assessing scaffold mechanics and degradation, immune response profiling, and evaluating long-term functional outcomes to cross into human therapies.
- Bioprinting systems must be able to operate beneath appropriate Good Manufacturing Practice (GMP) or closely aligned surroundings, as regulatory authorities will perform increasing scrutiny on results. Output must prioritize accuracy, safety, sterility, and ease of tracing.
- Authoring clinicians, biologists, and engineers from differing disciplines will be crucial as the focus shifts to the more complex aspects of organ repair and replacement.
- The price, availability, and eco-friendliness of the tech will also be factors: using optimized bioinks from renewable resources, low-cost materials, and waste tech that is accessible in low-resource areas for more efficient printing (for example, to treat ulcers and wounds).
- More intricate bioengineered tissues will expose ethical issues (patient cells, tissues, immune matching cross, iPS cells, and their donors) that must be addressed as in more complex bioengineered tissues.

## Conclusion

In general, the outcomes of this Research Topic paint a lively and fast-developing landscape. The advancements in bioink formulation, scaffold construction, modified matrices, dynamic printing, and scaffolds of clinical significance demonstrate that 3D printing and bioprinting are not laboratory curiosities. While major challenges still exist, particularly relating to scale, vascularization, regulatory compliance, and integration, the advances presented here indicate that most of these challenges can be overcome. We hope this Research Topic serves not only as a source of cutting-edge information to the researchers but also inspires them to accelerate the implementation of 3D printing and bioprinting technologies and techniques into practical medical treatments.

